# Engaging the broader community in biodiversity research: the concept of the COMBER pilot project for divers in ViBRANT

**DOI:** 10.3897/zookeys.150.2149

**Published:** 2011-11-28

**Authors:** Christos Arvanitidis, Sarah Faulwetter, Georgios Chatzigeorgiou, Lyubomir Penev, Olaf Bánki, Thanos Dailianis, Evangelos Pafilis, Michail Kouratoras, Eva Chatzinikolaou, Lucia Fanini, Aikaterini Vasileiadou, Christina Pavloudi, Panagiotis Vavilis, Panayota Koulouri, Costas Dounas

**Affiliations:** 1 Institute of Marine Biology and Genetics, Hellenic Centre for Marine Research, 71003 Heraklion, Crete, Greece; 2 Department of Zoology-Marine Biology, Faculty of Biology, National and Kapodestrian University of Athens, Panepistimiopolis, 15784, Athens, Greece; 3 Department of Biology, University of Crete, 71409 Heraklion, Crete, Greece; 4 Pensoft Publishers, Geo Milev Street 13a 1111 Sofia, Bulgaria; 5 Global Biodiversity Information Facility, Universitetsparken 15, DK-2100 Copenhagen, Denmark; 6 Hellenic Centre for Marine Research, 71003 Heraklion, Crete, Greece; 7 Department of Biology, University of Patras, 26504 Rio, Patras, Greece

**Keywords:** Citizen science, marine biodiversity, SCUBA diving, data collection and publication, sustainability

## Abstract

This paper discusses the design and implementation of a citizen science pilot project, COMBER (Citizens’ Network for the Observation of Marine BiodivERsity, http://www.comber.hcmr.gr), which has been initiated under the ViBRANT EU e-infrastructure. It is designed and implemented for divers and snorkelers who are interested in participating in marine biodiversity citizen science projects. It shows the necessity of engaging the broader community in the marine biodiversity monitoring and research projects, networks and initiatives. It analyses the stakeholders, the industry and the relevant markets involved in diving activities and their potential to sustain these activities. The principles, including data policy and rewards for the participating divers through their own data, upon which this project is based are thoroughly discussed. The results of the users analysis and lessons learned so far are presented. Future plans include promotion, links with citizen science web developments, data publishing tools, and development of new scientific hypotheses to be tested by the data collected so far.

## Introduction

### The interdisciplinary nature of biodiversity science and current problems

The Rio Earth Summit (1992) drew international concern to the global biological diversity loss and transformed the concept of biodiversity into a matter of public awareness and into an important issue in the political arena (Magurran 2004). The extent to which changes in biodiversity may induce reduction of ecosystem performance and of its potential to provide humankind with products and services still remains the focus of much scientific effort (Worm et al. 2006). The effects of these changes on the ecosystem’s goods and services may imply losses of several trillions of dollars forever (e.g. Costanza et al. 1997). These calculations have been made, however, without taking into account either those ecosystem functions to which no value was assigned (e.g. their ability to perform the biogeochemical cycles), nor the societal consequences caused for example by the lost jobs, especially in the current volatile global economy.

Perhaps, the major achievement after the Rio Summit was that it changed scientists’ views on ecosystem theory. The CBD (Convention on Biological Diversity 1993) forced scientists to consider multiple levels of biological organisation (e.g. genes, species, ecosystems) and an extended range of geographical or any other type of observational scales (e.g. from local to global) in which alterations may occur. These changes in scientific thinking brought to researchers, environmental managers, and policy makers the issue of the vast amount of data and information required to meet the CBD’s goals, such as monitoring and conservation of biodiversity at a global scale. However, there are two fundamental problems which seriously impede our efficiency in the collection of the datasets required to achieve the targets set by the CBD: the biodiversity crisis (e.g. Singh 2002) and the taxonomic impediment(e.g. Agnarsson and Kuntner 2007). The former problem refers to the decline of biodiversity resources and has emerged as one of the major economic issues of this century. Quantifying the change in biodiversity and the resulting impact on ecosystems’ goods and services for humankind is seriously hampered by the latter problem, that is, by the major gaps in our taxonomic knowledge (Lyal and Weitzmann 2004, Wheeler et al. 2004, Carvalho et al. 2005). A recent study by Mora et al. (2011) has estimated that ~8.7 million eukaryotic species exist globally, of which ~2.2 million are characterised as marine. As only 1.2 million species have hitherto been catalogued, this means that some 86% of the existing species on Earth and 91% of the species in the ocean still await description. Although the term “taxonomic impediment”refers to the discipline of taxonomy, the multidisciplinary nature of biodiversity implies that it adversely affects other disciplines such as ecology: the inability to accurately classify the organisms into species (/taxa) results in poor ecological datasets and conclusions based on them. Another dimension of this problem is that the population of the professional data collectors (e.g. taxonomists) is diminishing. Consequently, solutions should be sought along two directions: (a) to find ways to increase taxonomic efficiency and (b) to establish data collection programmes and networks.

### From conventional taxonomy to web-based “cybertaxonomy”

Descriptive taxonomy and classification of living organisms has its origins in Ancient Greece (Aristotle) and in its modern format dates back nearly 250 years, when Linnaeus introduced the binomial classification system still in use today. After almost 200 years of flourishing, the discipline is confronted by serious problems primarily because of the aged system used for its administration: The rules and conventions for descriptive taxonomy date back to the nineteenth century and the corresponding nomenclatural codes (e.g. zoological, botanical) that were developed in the mid 20th century, have not been updated to embrace modern information technology. Only very recently, the old tradition of communicating taxonomic acts through printed paper has started being replaced by approaches allowing electronic means (such as online-only journals) to publish scientific findings, as decided for example by the International Botanical Congress in Melbourne in July 2011 (Knapp et al. 2011). However, so far only the International Code of Botanical Nomenclature has incorporated these changes; for taxonomic acts in zoology, printed versions are still required. Crucial taxonomic information for the active functioning of the discipline, the type-material of each species, is still made available only through formal loans from museums and academic zoological/botanical repositories (Causey et al. 2004). In the twentieth century, taxonomy expanded towards modern disciplines such as genetics and phylogeny (Godfray 2002). The phenomenal explosion of sequence, genomic, transcriptomic, proteomic, metabolomic and other molecular disciplines, has largely been assisted by the achievements of computer science and internet technology (e.g. Johnson and Browman 2007). As a consequence, the rules for their functioning and the potential for their further development resulted in world-wide information facilities and projects/initiatives (e.g. the Consortium for the Barcode of Life – CBOL, http://www.barcoding.si.edu (Herbert et al. 2003, Stoeckle 2003), or the Global Biodiversity Information Facility – GBIF, http://www.gbif.org), launched as an international platform to aggregate and index occurrence data worldwide. Molecular classification has inevitably utilised computing power for the development of robust phylogenies and resulted in initiatives, such as the Assembling the Tree of Life initiative – ATOL, http://www.phylo.org/atol (Cracraft and Donoghue 2004). At the same time, taxonomy publishing has also been experiencing major developments in the past few years. Several important components of the Semantic Web, such as cross-linking, semantic tagging, data publication, data sharing, data aggregation, etc., have become ordinary components in the vocabulary of the biodiversity scientists (Penev et al. 2010a, b). Therefore, internet and web developments can profoundly assist current science to overcome the taxonomic impediment.

### Engaging a broader community in marine biodiversity research

Most of the ecological information and data are collected in the framework of temporally limited projects, simply because the collection costs are covered by the project funds. This trend commonly results in series of datasets that are predominately discontinuous or unevenly spread, geographically, temporally or ecologically. The latter becomes more obvious in the marine environment in which the collection costs are much higher than in the terrestrial realm due to the diverse and expensive floating means as well as the specific sampling gears and methods used. Several international projects which are targeted at continuous data collection from specific habitats have been launched in the last couple of decades. An exemplar project of this category is the NaGISA project (National Geography in Shore Areas; http://www.nagisa.coml.org/) which operates under the umbrella of CoML (Census of Marine Life, http://www.coml.org/). As the population of the professional taxonomists is diminishing, the mobilization of citizen scientists has become a key element to the success of the information and data collection process (e.g. Delaney et al. 2007, Hand 2010, Silvertown 2009, Trumbull et al. 2000). The implementation of citizen science in the marine environment currently faces two difficulties: (a) only the tidal zone can be approached by all citizens, and (b) the maximal depth safely reachable by recreational SCUBA divers is limited to 40 m. In the latter case, expensive diving equipment and certified training are required.

### Community development in web-based biodiversity data systems and the role of COMBER

COMBER (Citizens’ Network for the Observation of Marine BiodivERsity, http://www.comber.hcmr.gr) is a pilot project which has been initiated under the ViBRANT e-infrastructure and as part of this it taps into a suite of developments aimed at supporting virtual research communities in biodiversity science. ViBRANT is a European funded FP7 project (2010-2013) with the goal to provide an integrated framework of existing and newly developed services for managing biodiversity data. Scratchpads are the platform for these developments, and this platform is based on Drupal. Within ViBRANT the necessary links will be constructed to enable a free and usable data flow between Scratchpads and existing standardized taxonomic infrastructures (e.g. CBOL, EDIT platform, EOL, GBIF).

COMBER aims at engaging citizen scientists – that is, all persons interested in nature– in a coastal marine biodiversity observation network. It is currently operating in the Cretan (Greece) coastal environment with the potential to expand to the whole Mediterranean basin or any other European region. The activities have also been demonstrated in a few other coastal areas of the southern Aegean Sea. The basic characteristics of this pilot project are: (a) a web site which has been developed and functions as the main communication and promotion vehicle of the network, offering data-entry tools for collecting information which, at a later stage, are channeled to large data aggregators (e.g. GBIF) and publication media (e.g. PENSOFT); (b) a well-defined scientific hypothesis which has been formulated to be tested with the collected data; (c) a focus on fish species; (d) a suite of tools, such as a waterproof identification guide (see below), on-the-spot professional introductory lectures, underwater training, and demonstration of web site usage as well as data entry which are used to facilitate *in vivo* identifications by participating divers; (e) collaboration with two commercial diving centres in order to ensure operational safety and to explore the market development potential for the sustainable continuation of the initiative after the end of the project; (f) exploration of new services and tools to enhance the SCUBA diving and snorkeling services which are targeted towards the tourism industry.

## Material and methods

### Users, stakeholders, industry and market approach

The different categories of all the interested parties were identified during the design phase of the project: (a) a user is any person interested in participating in the activities of the project; this category includes people skilled to dive with a mask and a snorkel or certified SCUBA divers; (b) the main stakeholders identified so far are the diving centre instructors and owners, the directors of the tourist offices and the director of the Cretaquarium (HCMR); they were all approached and informed about the project, its activities and the potential it may create for the tourist industry and local markets; (c) the only industry involved is the tourist industry and its relevant markets which in this case are the services offered by the diving centres and by the Cretaquarium.

Potential participants were informed about the project through: (a) the website of the project; (b) an information desk in the Cretaquarium; (c) posters and leaflets which were distributed in the participating diving clubs and in the tourist information offices. Often, divers were approached directly before their dives in the diving centres and usually expressed interest in participation.

### Training and data collection

Fish species were chosen as a target taxon for the implementation of the pilot project since they are abundant and most frequently attract the attention and interest of the wide audience. The species observation and data collection was facilitated by usage of the commercial BIOWATCH underwater fish card (http://www.bio-watch.com). The underwater fish card (Dounas 2009, Dounas and Koulouri 2011) includes the forty most common fish species of the Mediterranean coastal environment and it differentiates them on the basis of morphological characteristics (e.g. body shape, fin morphology), colour pattern, and habitat. During the dive, each participant was equipped with a fish card which was used both to identify species and directly note down observations during the dive. For convenience, it was suitably modified to be attached on the diver’s buoyancy control device (BCD) with a rope and clips. In addition, small circles were drawn next to each species figure to assist the divers to quickly and accurately record their observations. Four abundance classes were assigned, following a geometric scale: (a) absence, indicated by a blank field; (b) 1–3 individuals, marked by a single bar; (c) 4–10 individuals, marked by two bars; (d) more than ten individuals, marked by three bars.

Training of participants in data collection and data entry was implemented as short seminars given by marine scientists. The seminars were divided into three parts: (a) Before the dive, participants followed a short (~15min) introduction on the data collection protocol, including how to distinguish target fish species using the underwater fish card and correctly record the observations; (b) During the dive, each scientist accompanied maximally 3 participants to continue training in fish identification and data recording, thus ensuring maximally possible accuracy. During the first 10–15 minutes of each dive the scientists pointed out various fish species and helped the participants in correctly identifying them. After this initial period, participants were encouraged to continue the data collection by themselves, however, the scientists were available for help all the time; (c) After the dive, a short de-briefing and discussion of possible questions followed. Participants were then introduced to the website, created an account, completed their diving profile (e.g. diving level, number of total dives), logged dive information (e.g. location, depth, visibility, air consumption) and recorded the observed species. Finally, participants were asked to complete a questionnaire targeted at the experiences gained through participation and the perception of the project. The questions included can be roughly divided into five categories: (a) motivation to participate (5 questions), (b) perception on the continuation of the project (1 question), (c) willingness to pay for a similar service in the future (1 question), (d) project design and implementation (4 questions), and (e) suggestions and comments (4 questions).

### Web developments and data management

COMBER uses Drupal (http://www.drupal.org), a free and open source Content Management System (CMS) as a software to perform all underlying functionality of the system. This allows full interoperability with ViBRANT and Scratchpads which are based on the same software. Many elements of the site, such as user management, profile creation, image galleries and discussion fora have been created using built-in features or readily available Drupal modules. Users can log into the site with their Facebook account, a valuable feature to strongly facilitate the registration process on the site. Registered users can continue to contribute data after participation in the seminars, use the diving log to keep track of their dives and species observations, upload photos of fish species and discuss various topics in the discussion fora. A competitive element is introduced by a five-star ranking system indicating the activity level of the user – the more dives with fish observations are contributed to the system, the higher the user ranks in a “Top contributors” list, thus providing a playful incentive to contribute (see relevant paragraph below).

## Results

### Principles and implementation

**Principles**

The COMBER pilot project has been designed according to five fundamental principles: (a) Diving safety, ensured by involving two certified diving centres in the project which were responsible for the strict adherence to safety rules; (b) Simplicity: this principle refers to the underwater observation protocol and is extremely important, especially for non-professional recreational divers, because the diving process itself contains many elements requiring the divers’ concentration (buoyancy control, pressure equalising, air consumption, adjusting to swimming underwater, monitoring depth and dive time to calculate the dive profile and avoid dangers of decompression sickness and control of diving equipment). Therefore, an additional activity such as the observation and recording of the fish species and their relative abundance on the fish card definitely introduces an additional concern which may easily turn into stress. The data collection protocol has thus been designed in a very straightforward way to require as little effort from the divers as possible; (c) Efficiency: this principle refers to the accuracy of the data collected by SCUBA divers without experience in fish identification. The fishcard focuses on easily recognisable characteristics to identify fish species. Colour and patterns are the most easily used characteristics. However, due to the progressive absorption of wave lengths of the light with depth, most of the colours except for green and blue tones tend to disappear after ca. ten metres depth. Therefore, the briefing before diving focuses on body shape and colour patterns which are not lost, and the training is continued underwater by observing living animals. This transition is very important to train the divers in how to work most accurately and also to provide them with some sense where to search and in which habitats certain species are to be found; (d) Interdisciplinarity: many scientific disciplines are actively involved and interrelated in this experiment: taxonomy, ecology, statistics, sociology, economics, education; (e)Sustainability: all of the above interrelated disciplines serve the same dual goal: to involve citizen scientists in order to produce reliable data and information and to sustain these activities for as long as possible through the development of the relevant network, goods, and services.

**Rewarding for all involved parties**

The users/contributors of the COMBER activities and the project infrastructure are rewarded by: (a) a free BIOWATCH fishcard after their participation to the project; (b) the COMBER website, which – besides offering tools to keep an electronic dive log – provides facilities to upload annotated photos and discuss with other divers in a social networking environment and automatically accredits “contribution stars” to the divers according to their activity level (number of dives); (c) the association of their name with the information and data from the moment they submit their data, ensuring full credits for their work in any upcoming publication which uses these data.

**Data policies and management**

The pilot project closely follows ViBRANT’s policy on the management of intellectual property. The concept of “Open Science” is adopted by COMBER as an overarching principle. In short, this concept implies the free/open software use under the Creative Commons movement. Clear documentation of the methodology used and of the data and results extracted is centrally placed in this concept. The intellectual rights of the information and data submitted by the user always stay with the user and allow him/her to get flexible rights for reuse. All the relevant statements and legal conditions regulating this policy are published on the web page of the pilot project. Any application, including software, source code, is free for use (GNU General Public License). Any other content uploaded on the COMBER web page, such as training courses, literature references and resources, images, videos, etc., are also distributed under a Creative Commons license and hence free for use by any user, provided that credits are given upon re-use of data.

**From concept to implementation**

**Figure 1.**
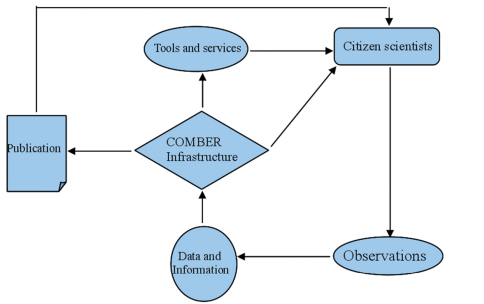
Schematic representation of the basic components of the COMBER project.

The concept of the basic components of COMBER as well as the activities and information flow is shown in Figure 1. The central component of the project is the COMBER web infrastructure, which consists of a web-accessible front end for dissemination of information and data entry interfaces, as well as data management and storage services on the back end. Contrarily to these virtual tools, the component of tools and services currently refers to those provided on the spot, such as the underwater fish card, the SCUBA diving equipment, and the training by professional scientists. However, this part will eventually include commercial services to raise funds for the sustainability of the project after the end of the ViBRANT funding. Citizen scientists make direct use of the latter component during their dives and they are closely linked to the former component through the use of the web infrastructure and the virtual tools and services, including the reward system. The component of the “observations” comprises the actual species observations by the divers, which are recorded during their dive time. This data collection is an essential step in the process and therefore an important component of the project. The species identity data, as well as information on the diving profile of the diver, answers to the questionnaire, diving location, accompanying HCMR scientists and dive masters, weather conditions and typical diving information (tank charge, depth, duration), are then all uploaded to the electronic infrastructure of COMBER and are always associated to the diver’s name. The entire set of the submitted data are the intellectual property of the contributor (but free for use under a Creative Commons license, see below); by associating the name to the data it is ensured that the contributor receives full credits for his work in future publications.

### User analysis

**Identity of the participants**

**Figure 2.**
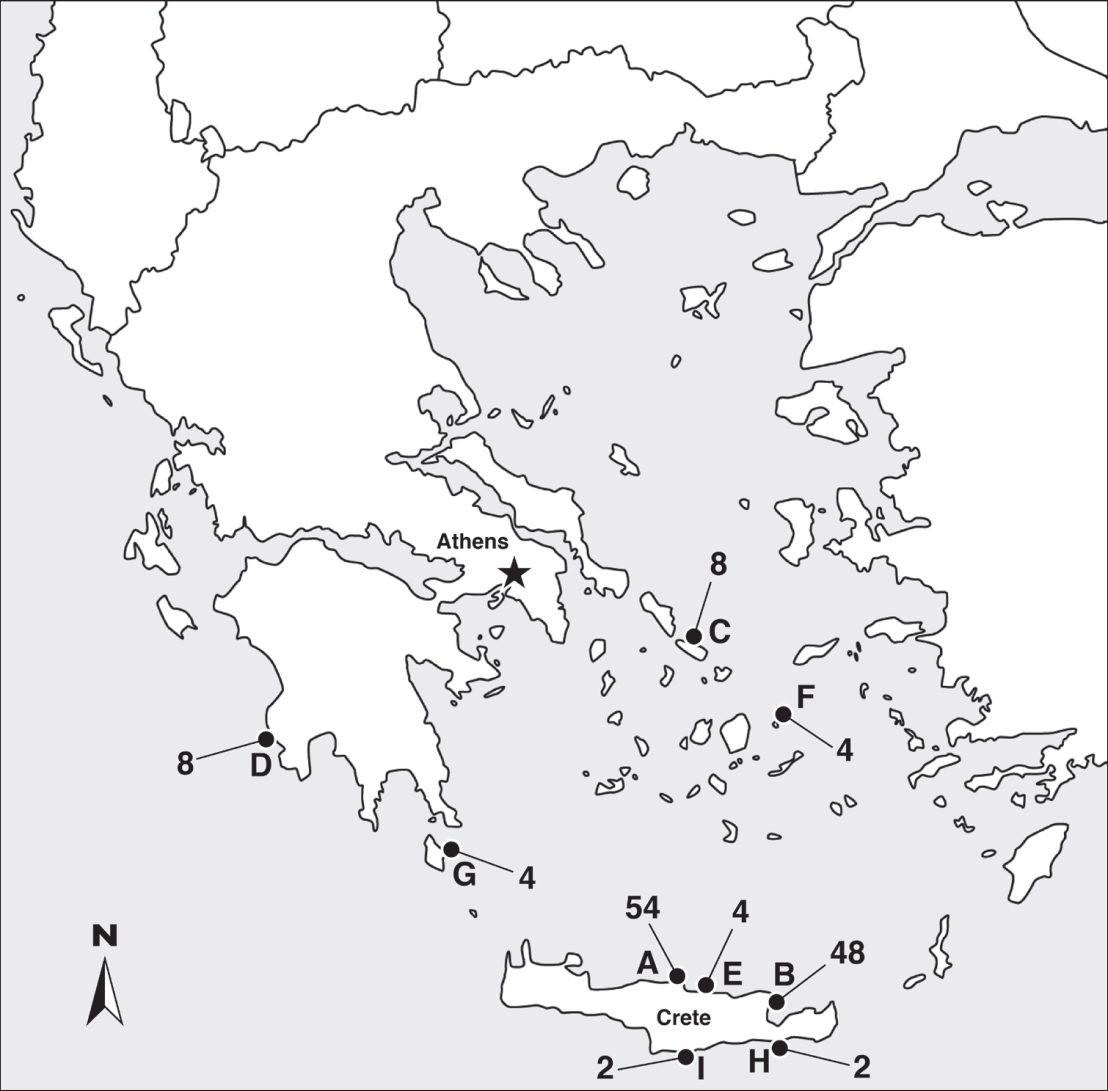
Map of observation sites: **A** = Lygaria **B** = Agios Nikolaos **C** = Tinos **D** = Pylos **E** = Hersonissos **F** = Donousa **G** = Kythira **H** = Ierapetra **I** = Tripiti. The two diving clubs where the project was conducted under supervision of the scientists are based in Lygaria and Agios Nikolaos (A, B). Numbers refer to dives or snorkel trips.

During the two months of the project (July–August 2011), 48 users (excluding the four supervising scientists) participated in the project. Twenty of the users contributed data from more than one dive or snorkeling trip and thus expanded the sampling area to several other locations in Greece (Figure 2). In total, 1,879 species observations were recorded during 95 dives and 39 snorkelling trips.

Participants came from ten countries, with the majority (42%) coming from Greece, followed by the United Kingdom and the Netherlands (12% each). The majority (70%) of the participants held a basic-level diving certificate (PADI Open Water / Advanced Open Water, CMAS *), 12% held an advanced certificate (PADI Rescue Diver, CMAS **) and 16% held a professional diving license. However, half of the divers had an advanced diving experience (>30 dives), independent of their certificate. Most of the participants already had certain knowledge about marine organisms (72% declared they had advanced (36%) or basic (36%) knowledge about marine organisms, while 28% declared they had no knowledge at all). The genders were unevenly distributed (64% male, 36% female), but all age groups were present (21–55 years), with a slight dominance of 20–30 year old (39%) and 40–50 year old (30%) persons (30–40 years old: 18%, >50 years old: 13%). Two (independent) criteria can be applied to the profile data, each one separating the participants into two equally sized groups: I. age/profession: (a) a group of young (<30 years old) local participants, most of them biology students; (b) persons over 35 years old, mostly male, none of them pursuing a profession related to biology, but almost all of them with an academic education. They originate from various countries (thus many being tourists). Both the diving level and the knowledge of marine organisms were heterogeneously distributed in both groups; II. diving skills/knowledge of marine organisms: (a) a group which had little diving experience (<30 dives); 82% of these participants had basic or no knowledge of marine organisms; (b) a group of experienced divers (>30 dives); here 64% claimed they had an advanced knowledge of marine organisms.

**Behaviour, motivation and perception of the project**

During the dives, the experience in diving was a major factor contributing to underwater behaviour and data collection. Inexperienced divers (with <30 dives) moved slowly, needed more time to observe and identify the fish, asked questions more frequently and needed more supervising during their dives. The major factor which generally influenced their behaviour was the effort spent to control their buoyancy and equipment, thus it was harder for them to focus on diving and observing at the same time. Experienced divers moved in a more efficient way, they needed less time to observe and identify species and to collect information, they observed fish in different habitats (e.g. under rocks, in the water column above them) and needed much less supervising attention by the scientists. From the observations of the accompanying scientists, a general trend for increased quantity and quality of data with an increasing number of dives could be discerned, the validity of which is currently tested in ongoing analyses of all data.

The results from the questionnaires concerning the motivation for participation and participants’ perception of the project (answered by 25 users) can be divided into three broad categories: a) Identification process: The majority of the divers (64%) declared that some fish were easy to recognise but they had doubts about the validity of their results, while the remaining persons had no difficulties in identifying species. However, 90% of the participants found the short seminars before the diving helpful and claimed that by using the fish card only, they would have had problems to identify the species; b) Motivation: A large part of the participants (64%) had never participated before in any kind of volunteering work concerning nature conservation or observation. However, 28% are actively engaged in volunteer projects and 8% had already participated in similar projects but are not regularly engaged. Most of the divers participated because they appreciated the feeling of contributing and thus being useful for science and being part of an international network (48%) and because they like gaining new knowledge about nature (20%). Only a small percentage of them participated because their friends or dive buddies wanted to participate (8%) or simply out of curiosity (14%). The majority (84%) claimed they would continue contributing data on future dives, a minority stated that they were not interested, or they would like to but would probably lack motivation without instructors around (16%); c) Overall perception of the project: Both the project idea and its implementation were generally judged positively. On a scale from 1 (“did not like it”) to 5 (“liked it very much”), 96% rated both the project idea and its implementation to be good or very good, however, the implementation part was not always scored with full marks and participants provided valuable suggestions for improvement, most of them asking the organisers to: (a) offer more detailed introductory seminars about marine biodiversity and to make identification underwater easier; (b) provide online material (presentations, photos, videos, quizzes); (c) include more fish species and other taxa (e.g. sponges, mollusks) and (d) to better promote the website (through higher ranking in search engines, Facebook and Twitter). The project had a strong impact on the participants’ perception of biodiversity: 84% declared that they now see the underwater world with different eyes, only 16% claimed that the participation left no impression on them. This is reflected in the answers to the free-text questions concerning what participants liked most or what left an impression on them: 72% stated that they appreciated learning more about the marine life and that being able to differentiate species (and thus the greater diversity) made diving a richer experience. The actual diversity of life that they were not aware of before participation left a strong impression on many participants, but there was also a positive perception of experience of citizen science: divers were impressed by the difficulties of identifying species and data collection and thus the difficulties of conducting science and they felt a personal reward through their contribution to data collection. Overall, the project was highly appreciated and 80% of the participants declared they would be willing to even pay for a similar commercial course (e.g. a “marine biodiversity diver” course).

The major groups that were identified among participants were also reflected in their answers to the questions concerning the perception of the project. Of the persons who had no problems with the identification of fish, 63% had a good diving experience (>30 dives), while within the group of persons doubting their results, the experienced divers accounted for only 26%. Generally, the experienced divers also showed a higher willingness both to continue observations on their own (100% of the experienced divers and 71% of the non-experienced divers would like to continue data contribution), and to pay for a commercial offer (88% of the experienced, 78% of the non-experienced divers). Furthermore, people with an existing knowledge of marine life found the identifications easy, often had previously participated in volunteering projects and appreciated the ability to become a part of a scientific network and to contribute to science and knowledge creation. This group consisted of many local people, often young biology students; they expressed interest in more detailed seminars, in expanding the functionality of the website and in continuing the observations.

## Discussion

### Lessons learned

**Particular features of the industry and its associated markets**

Tourism is among the most prominent economic sectors in Greece, with an average annual contribution of more than 15% to the GDP which shows a constantly increasing rate in the recent years, approaching the 20% in 2011. Greece welcomed over 19.3 million tourists in 2009, a number which was further raised in the following years (http://en.wikipedia.org/wiki/Economy_of_Greece). This sector is very important for the country’s labour force and particularly for the island of Crete. A number of markets are associated with the tourism industry such as accommodation, transport, and recreation, which can potentially be positively affected by the proposed pilot project. However, there is still much uncertainty whether these markets follow the general trend for the industry. The recreation market, to which SCUBA diving services belong, is not directly associated with the tourism trend since it appears to have its own idiosyncratic dynamics. For example, during the current year in which tourism has been raised in Crete by 15% over the high season in comparison to last year, the SCUBA diving services sales dropped by a factor which reached 30%, at least as reflected in the accounting books of the collaborating diving clubs. This might relate to the fact that tourists visiting the island increasingly prefer to book their holidays in hotels offering “all-inclusive” accommodation and rarely participate in recreational activities not included in the pre-paid packages. This uncertainty has to be taken into account particularly when projections are made in a volatile economic environment.

**Homogeneity of the provided services and heterogeneity of the users**

Since international diving safety regulations do not allow for much variation in diving protocols, the diving process during the data collection is relatively homogeneous, despite a large variation in locations, habitats and species communities. On the other hand, there is a remarkable heterogeneity in divers’ attributes such as their skills, interests, expected rewarding, and repetitiveness of the dive, to cite a few among others. This mismatch between the diving process and the divers’ attributes may discourage many recreational divers, especially those who are at the beginners’ stage. The pilot project on the other hand, offers some positive arguments which, if correctly communicated, can be instrumental in increasing the number and frequency of the dives. This is simply because COMBER provides an alternative diving approach through which the divers can: (a) learn about the marine environment and its life; (b) contribute to the internationally recognised goal of marine biodiversity monitoring and conservation; (c) be rewarded for their involvement in the pilot project in multiple ways; (d) have fun in a team of other divers.

**Necessity of the “guided” approach and correction plans**

One of the most important lessons learned so far is that the supervising and guidance of the COMBER dives is instrumental for the success of the project. This guidance is implemented at all the three stages of the dive (before, during, after). The divers need some initial information on the pilot project before they start working underwater, such as the aim, the means, the expected results, the effort required by them, the target organisms, the way they have to work, the responsible bodies and people, and extra safety measures. All participants welcomed the guidance provided during the first ten to fifteen minutes of the dive in order to be introduced to fish identification and data collection in the field and to get an initial feedback on the accuracy of their observations. After this short period the divers generally seemed more confident with their identifications during the remaining dive time, although several of them usually kept requiring assistance. During the debriefing stage, the divers posed additional questions on some doubtful observations, on data entry through the web interface and on the continuation of their effort in the future. In most cases, discussions with the scientists and consultation of field guides allowed divers to critically assess and correct their own observations before entering them into the system, thus entering the “questioning” phase of their observation which is at the core of the scientific approach: seeking for the truth in their observations by using certain scientific criteria which in this case are taxonomic and, to a lesser degree, ecological characters. The latter has been specifically designed in order to avoid mis-observations leading to failure in the collection of reliable data, as has been observed in similar recent attempts (Goffredo et al. 2010).

### The way forward

**Future plans and promotion**

The engagement of the broader community is a big challenge not only for the project itself but also for the marine biodiversity discipline in general. It can be broadly regarded as a significant trade zone between science, on the one hand, and society, industry, and markets, on the other. The cornerstone on which this trading zone must be built is the sustainability of the activities to both these ends. Economically healthy and sustainable activities may also serve the production and publication of reliable datasets (see also next paragraphs) needed for the study, monitoring, and conservation of the marine biodiversity while the latter also raises the concern of society for healthy and productive ecosystems.

The project has initiated the efforts in order to identify the major stakeholders and the industry and relevant markets involved. However, for the sustainability of the activities it is also important to identify the relevant target groups that may play a crucial role in the project. Taking into account the results of the questionnaire, the future expansion of the project should be developed into two different directions, aiming at two major target groups: a) a more commercially-oriented offer for experienced divers (both tourists and locals), with more comprehensive and detailed seminars, allowing them to obtain an internationally recognised diving certificate (“marine biodiversity diver”), and b) focusing on the development of local “nature clubs” which are targeted at motivated, nature-loving persons living in the area, allowing them to regularly contribute, to engage themselves in nature conservation and to meet other people with similar interests. This target group could include (biology) students, local (amateur) divers and members of other nature clubs (such as hiking or photography groups) or any other interested person.

**How to use the data (from pre-treatment to scientific hypothesis testing including cleaning)**

Information and data must be corrected before they are subjected to scientific analysis and hypothesis testing. This process must also follow certain criteria based on specific assumptions. The basic assumption is that the fish species recorded by the professional scientist who is supervising the dive can be used as the first criterion to identify outliers in data collected by the divers. Additional criteria may be: (a) species which are not recorded by the scientists in any of the dives at a specific location should not be included in the datasets collected by the divers; (b) broad categories of depth or habitat (e.g. hard and soft substrates, seagrass meadows) can be another criterion following the same approach as above; (c) the same criteria apply also for the abundance classes records.

The next step after data cleaning is their use (and re-use) in testing the scientific hypotheses. This is still open to discussions within the ViBRANT consortium. However, the aim of the pilot project is to examine whether the data collected by the divers are suitable for biodiversity monitoring needs. Recent biodiversity measures, based on species relatedness such as the taxonomic distinctness (e.g. ﻿Warwick and Clarke 2001), could provide the concept to formulate and test the scientific hypothesis: whether the fish species lists collected by the divers are random samples from the regional species inventory. The relevant indices of the average taxonomic distinctness (Δ^+^) and variation in taxonomic distinctness (Λ^+^) can be used as the statistics to test the hypothesis.

**Data publishing horizons**

One of the key general concepts of the ViBRANT project is to provide an e-infrastructure to facilitate maximum possible automation of the whole process of handling taxonomic data, from the collection through data management and analyses, to the stage of publication, indexing and preservation. The ultimate goal of the pilot project is to create the network of the marine biodiversity citizen scientists and also the electronic infrastructure needed for the uploaded datasets to be channeled to all interested parties, such as global biodiversity species registries (e.g. GBIF, OBIS, etc.), and published by electronic publication media, using advanced data publishing technologies. Such a technology was currently launched by the “data paper” project by GBIF and PENSOFT Publishers. According to the concept (see Chavan and Penev, in press, and Penev et al. 2011 for a detailed description), occurrence datasets and/or taxon checklists can be uploaded through the Integrated Publishing Toolkit of GBIF (IPT) (http://ipt.pensoft.net/ipt/) in accordance with the Darwin Core mapping standards. During the upload the data author is requested to fill in extended metadata descriptions, based on the Ecological Metadata Language (EML). Metadata files include such important elements such as data authors, taxonomic and geographical coverage, project description, institutional support, data storage and software management, intellectual property rights and so on. After metadata are described, the author can generate a “data paper” manuscript from them, just by pushing a button. The manuscript is submitted to a scholarly journal and undergoes standard peer-review process. In case of acceptance, the author inserts the necessary corrections or additions recommended by the reviewers in the metadata on the IPT and then generate the revised manuscript again by pressing a button.

The data paper concept and associated tools were launched to provide incentives for data collectors to publish their data in a proper way, that is: (a) through enriched metadata description, and (b) indexing and collation of the data themselves within large international infrastructure, in this case, the GBIF data portal. The data paper will provide an opportunity for data collectors to be credited for their efforts and will open perspectives for a future collaboration with data authors having published similar types of data.

One important feature of the IPT with far-reaching consequences for biodiversity data publishing is the option for an easy creation of Darwin Core archives. The Darwin Core Archive (DwC-A) is an international biodiversity informatics data standard and the preferred format for publishing data through the (GBIF) network. The format is defined in the Darwin Core Text Guidelines. Darwin Core is no longer restricted to occurrence data, and together with the more generic Dublin Core metadata standard (on which its ideas are based), it is used by GBIF and others to encode metadata about organism names, taxonomies and species information. In addition, the whole set of data associated with the occurrence dataset, such as environmental measurement, habitat descriptions etc., can be deposited at the Dryad Data Repository (http://www.datadryad.org). Dryad provides a simplified metadata interface, however it assigns DOI numbers to each data file within a data package and to the data package as a whole. In addition to preservation and storage, Dryad also provides a workflow and standards that allow data to be cited in case they are used in future analyses, alone or with other data.

The current volume offers two exemplar papers that demonstrate the data publishing workflow described above (Faulwetter et al. 2011; Lambkin and Bartlett 2011). Both papers published data through (a) PENSOFT’s GBIF IPT, (b) Dryad Data Repository and (c) DwC-A supplementary files associated with the articles and downloadable from the journal’s website.

**Relevant web infrastructure developments**

GBIF has initiated a community driven project called the ‘Nodes Portal Toolkit’ that should enable communities to deploy, maintain, and extend biodiversity data portals. The project should provide an easy way for communities to start web based biodiversity data information systems with a link to the GBIF infrastructure. The GBIF Nodes Portal Toolkit will be Drupal-based, as this will allow for the integration of already existing modules. This informatics platform will also allow community development of new modules with extended functionalities for web-based biodiversity data information systems. The first version of the Nodes Portal Toolkit will be built around Scratchpads, linking well with developments in ViBRANT. A second version will have extended functionalities, such as a tool for displaying geographical distribution maps of species, similar to what is currently displayed in the OBIS data portal. We expect COMBER to become in the coming years fully integrated with the developments in ViBRANT and the GBIF Nodes Portal Toolkit, offering interested parties a ready-made installation file allowing them to set up and deploy their own citizen-science portals without prior technical knowledge.
